# Personalized radiotherapy of brain metastases: survival prediction by means of dichotomized or differentiated blood test results?

**DOI:** 10.3389/fonc.2023.1156161

**Published:** 2023-04-11

**Authors:** Carsten Nieder, Nicolaus H. Andratschke, Anca L. Grosu

**Affiliations:** ^1^Department of Oncology and Palliative Medicine, Nordland Hospital, Bodø, Norway; ^2^Department of Clinical Medicine, Faculty of Health Sciences, UiT – The Arctic University of Norway, Tromsø, Norway; ^3^Department of Radiation Oncology, University Hospital Zurich, University of Zurich, Zurich, Switzerland; ^4^Department of Radiation Oncology, Medical Center, Medical Faculty, University Freiburg, Freiburg, Germany

**Keywords:** brain metastases, prognostic model, score, biomarkers, tumor markers

## Abstract

**Background and objectives:**

The validated LabBM score (laboratory parameters in patients with brain metastases) represents a widely applicable survival prediction model, which incorporates 5 blood test results (serum lactate dehydrogenase (LDH), C-reactive protein (CRP), albumin, platelets and hemoglobin). All tests are classified as normal or abnormal, without accounting for the wide range of abnormality observed in practice. We tested the hypothesis that improved stratification might be possible, if more granular test results are employed.

**Methods:**

Retrospective analysis of 198 patients managed with primary whole-brain radiotherapy in one of the institutions who validated the original LabBM score.

**Results:**

For two blood tests (albumin, CRP), discrimination was best for the original dichotomized version (normal/abnormal). For two others (LDH, hemoglobin), a three-tiered classification was best. The number of patients with low platelet count was not large enough for detailed analyses. A modified LabBM score was developed, which separates the intermediate of originally 3 prognostic groups into 2 statistically significantly different strata, resulting in a 4-tiered score.

**Conclusion:**

This initial proof-of-principle study suggests that granular blood test results might contribute to further improvement of the score, or alternatively development of a nomogram, if additional large-scale studies confirm the encouraging results of the present analysis.

## 1 Introduction

Brain metastases should not be regarded as universally fatal cancer manifestation anymore (1–4). Even if survival outcomes of less than one year are still common, especially in patients with large burden of extracranial metastases who lack effective systemic treatment options (5), survival beyond 5 or even 10 years can be achieved in a minority of patients (6–8). Better understanding of factors explaining these survival differences has led to prognostic scores, which might aid clinicians who have to choose between vastly different treatment options. The latter include surgical resection, radiosurgery, other radiotherapy, systemic treatment and best supportive care, if other approaches are unlikely to prolong survival or improve quality of life (1–4).

Prognostic scores come with often unique advantages and disadvantages and are not expected to provide perfectly accurate survival predictions (9, 10). Gradual improvement of scores, e.g. diagnosis-specific graded prognostic assessment (DS-GPA), has long been a focus of dedicated research groups (11, 12). The validated LabBM score (laboratory parameters in patients with brain metastases) represents one of the DS-GPA’s competitors and has been studied in Austria, Switzerland and Norway (13, 14). It covers all cancer types and is solely based on easy-to-evaluate blood test results, allowing for rapid clinical implementation also in busy practices. LabBM incorporates five inexpensive blood tests (serum lactate dehydrogenase (LDH), C-reactive protein (CRP), albumin, platelets and hemoglobin), which indirectly reflect disease burden and its impact on inflammatory processes in the body. Normal blood test results are expected in case of limited disease extent. In contrast, multiple extracranial metastases and large overall tumor burden typically cause abnormal test results. The latter were simply dichotomized (normal/abnormal) in the original and validation LabBM studies. Elevated LDH led to 1 point, elevated CRP to 1 additional point, low hemoglobin, albumin and platelets each added 0.5 points to the LabBM score. A maximum sum of 3.5 points can be achieved and patients are assigned to one of three prognostic groups (best survival: 0-1 points, intermediate: 1.5-2 points, short survival: 2.5-3.5 points). As repeatedly shown, even in patients irradiated for non-brain metastases (15), the LabBM score performs well. It shares a limitation with other scores, i.e. the relatively large subgroup of patients with intermediate prognosis.

An important question has not been evaluated in the previous studies: do dichotomized blood test results provide optimum information or should we account for magnitude of deviation from normal values? It cannot be taken for granted that, e.g., a LDH level of 275 U/L provides the same prognostic information as a level of 550 U/L or 1,100 U/L. The purpose of the present study was to test the role of non-dichotomized blood test results in comparison to the original version of the LabBM score.

## 2 Materials and methods

### 2.1 Study population and data collection

This initial study (proof-of-principle or pilot study) was performed in a limited, but homogeneously treated population to minimize confounding factors, and speed up the process of signal detection, i.e. results that would justify large-scale analyses. A pre-existing database (14) with dichotomized blood test results was expanded to include the actual level of deviation in patients with abnormal results. All patients had received palliative whole-brain radiotherapy (WBRT, 10 fractions of 3 Gy, no preceding surgery or other brain metastases therapy) for multiple brain metastases at Nordland Hospital (time period 2007-2021, consecutive patients). Systemic treatment and salvage for progressive brain metastases, e.g. radiosurgery, were administered as indicated. The blood tests were part of routine oncological assessment approximately 1-2 weeks before WBRT. Normal values: hemoglobin 11.7-15.3 g/dl (females) and 13.4-17.0 g/dl (males); platelets 130-400 x10^9^; albumin 34-45 g/l; LDH <205 U/l; CRP <5 mg/l.

### 2.2 Statistical analysis

The original LabBM score (dichotomized blood test results) was calculated as outlined in the Introduction. Then, the underlying, now non-dichotomized blood test results were analyzed in univariate Cox regression analyses for continuous variables to test their prognostic impact. Next, optimum cut-off points were identified to group the results (maximum 4 strata per blood test, log-rank tests for actuarial overall survival curves, focus on best possible separation of the curves). Actuarial overall survival was calculated (Kaplan-Meier method) from the first day of WBRT, including also discontinued courses of WBRT. Date of death was known in almost all patients, while 5 were still alive at the time of analysis. Their median follow-up was 45 months (minimum 17 months). After these univariate analyses, the optimally stratified blood test variables were entered into a multivariate forward stepwise Cox regression analysis. Test results with significant impact on survival were then employed to assign a modified LabBM score, adhering to the original strategy described by Berghoff et al. (13) (0.5 or 1 point, depending on a factor’s regression coefficient B, or in other words impact on survival). P-values ≤0.05 were considered statistically significant. Analyses were performed in SPSS 28, IBM Corp., Armonk, NY, USA

### 2.3 Ethical statement

All procedures performed in studies involving human participants were in accordance with the ethical standards of the institutional and/or national research committee and with the 1964 Helsinki declaration and its later amendments or comparable ethical standards. As a retrospective quality of care analysis employing an established database, no approval from the Regional Committee for Medical and Health Research Ethics (REK Nord) was necessary. This research project was carried out according to our institutions’ guidelines and with permission to access the patients’ data. Written informed consent was received from all patients.

## 3 Results

**Figure 1:**
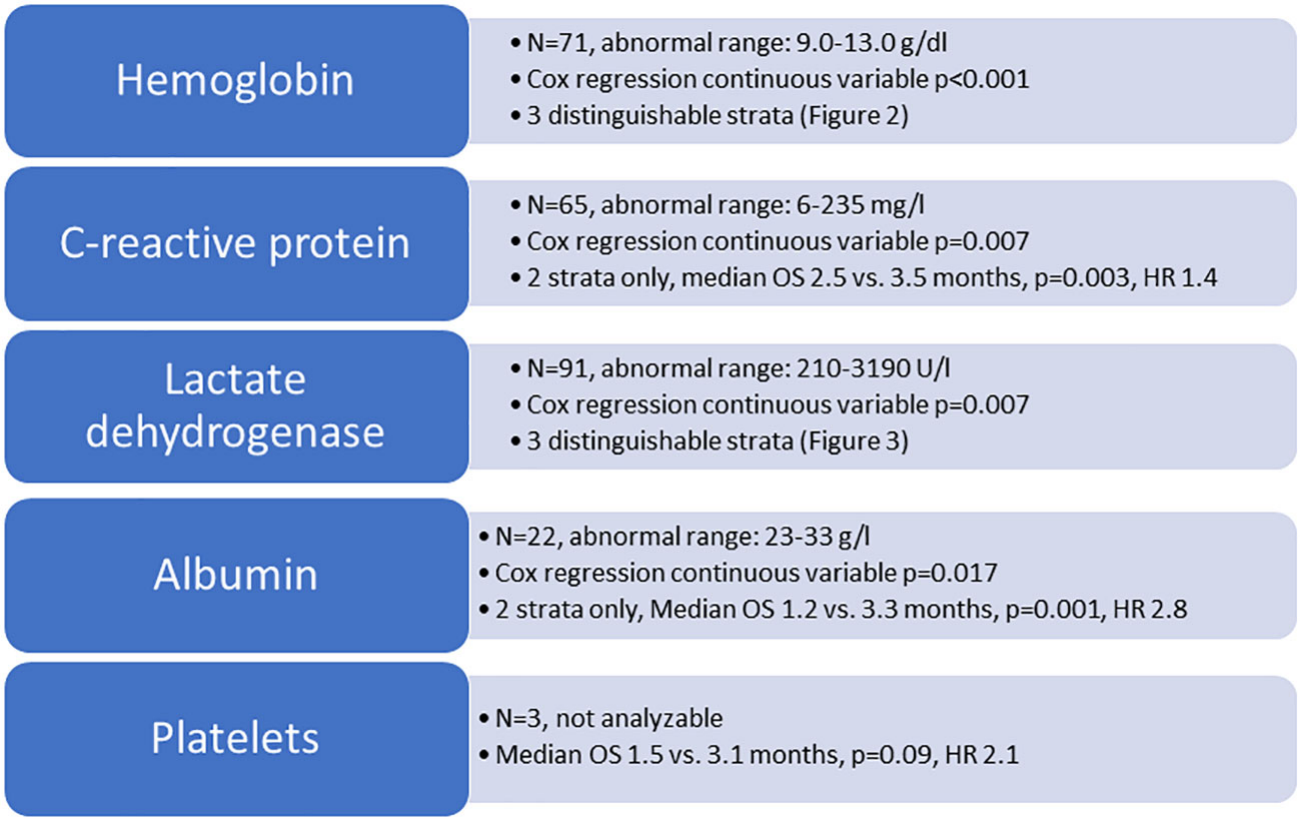
Overview of blood test analyses. OS, overall survival (Kaplan-Meier analysis, log-rank test); HR, hazard ratio.

**Figure 2:**
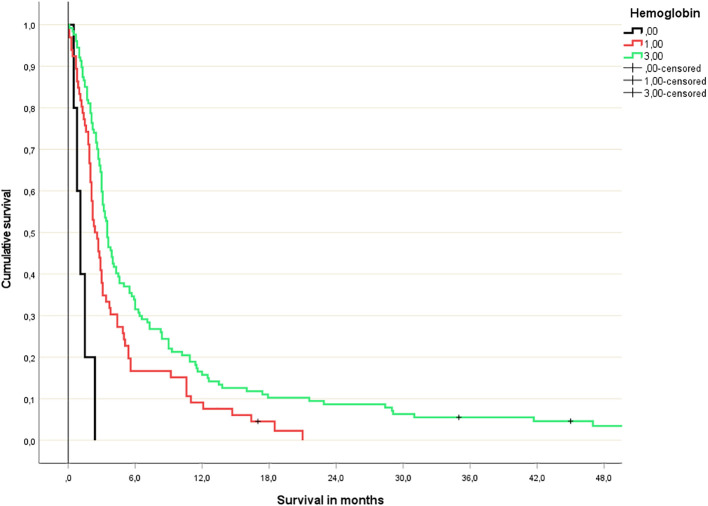
Hemoglobin-based 3-tiered survival prediction (Kaplan-Meier analysis; n=5 (hemoglobin <10 g/dl), 66 (hemoglobin 10-13 g/dl) and 127 (normal hemoglobin), respectively; median survival 1.1, 2.4 and 3.5 months, respectively; p=0.03 or better for all comparisons; HR 3.2 and 1.5, respectively).

**Figure 3:**
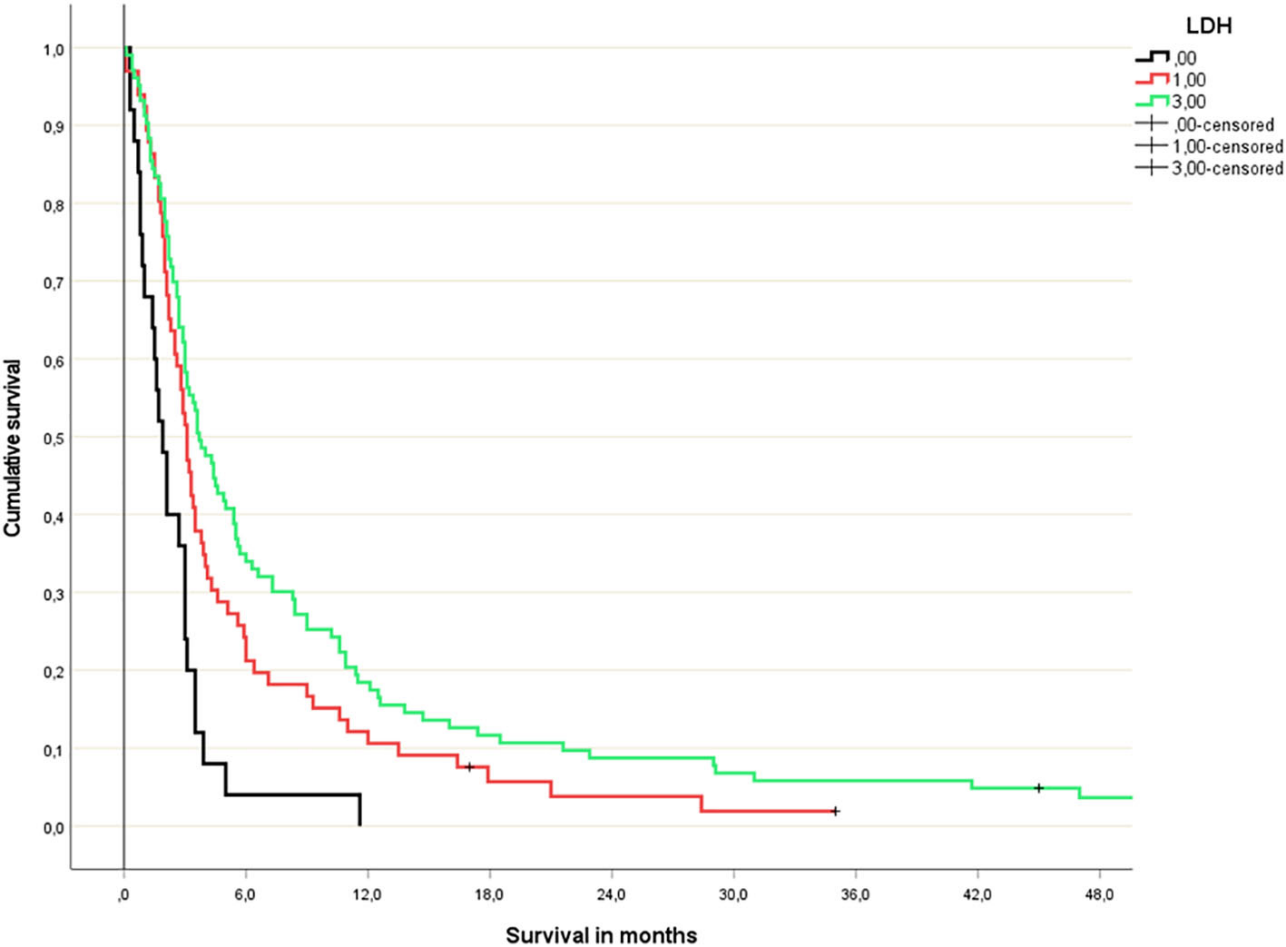
Lactate dehydrogenase-based 3-tiered survival prediction (Kaplan-Meier analysis; n=25 (LDH >400 U/l), 66 (LDH 210-399 U/l) and 103 (normal LDH), respectively; median survival 1.9, 3.1 and 3.7 months, respectively; p=0.04 or better for all comparisons; HR 1.9 and 1.2, respectively).

The study included 198 patients (193 death events) whose baseline characteristics are shown in **Table 1**. Not all patients had complete blood test results available. Due to low numbers, continuous variable Cox regression analysis was not feasible for patients with low platelet count (n=3). **Figure 1** shows the results of the analyses for the remaining 4 blood tests. For two of these (albumin, CRP), discrimination was best for the original dichotomized version (normal/abnormal). For the two others (LDH, hemoglobin), a three-tiered classification was best (**Figures 2**, **3**).

**Table 1:** Patient characteristics (n=198).

Baseline parameter	Number	Percent
Non-small cell lung cancer	83	42
Small cell lung cancer	8	4
Breast cancer	42	21
Malignant melanoma	25	13
Renal cell cancer	14	7
Colorectal cancer	10	5
Esophageal cancer	6	3
Bladder cancer	4	2
Other or unknown primary tumors	6	3
Extracranial metastases	168	85
No extracranial metastases	30	15
Controlled primary tumor	128	65
Uncontrolled primary tumor	70	35
Female gender	112	57
Male gender	86	43
Systemic therapy after whole-brain radiotherapy	93	47
LabBM score >2	18	9
LabBM score 1.5-2.0	55	28
LabBM score 0-1.0	102	52
Unknown LabBM score	23	12
Low albumin	22 (3 missing)	11
Low hemoglobin	71 (0 missing)	36
Low platelets	3 (14 missing)	2
High C-reactive protein	65 (19 missing)	33
High lactate dehydrogenase	91 (4 missing)	46

**Figure 4:**
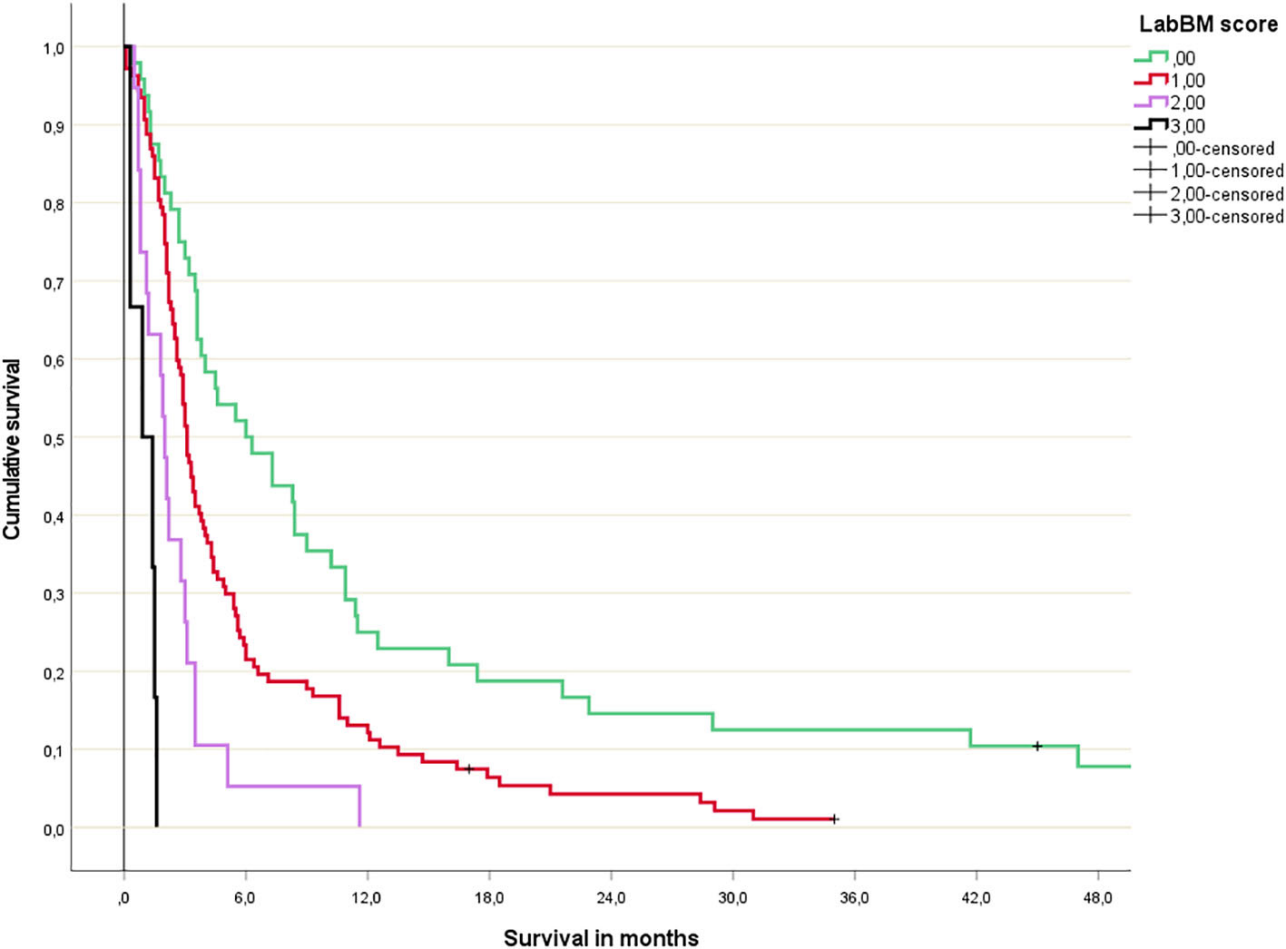
Modified LabBM score 4-tiered survival prediction (Kaplan-Meier analysis; for further details please see **Table 3**; p=0.01 or better for all comparisons).

**Figure 5:**
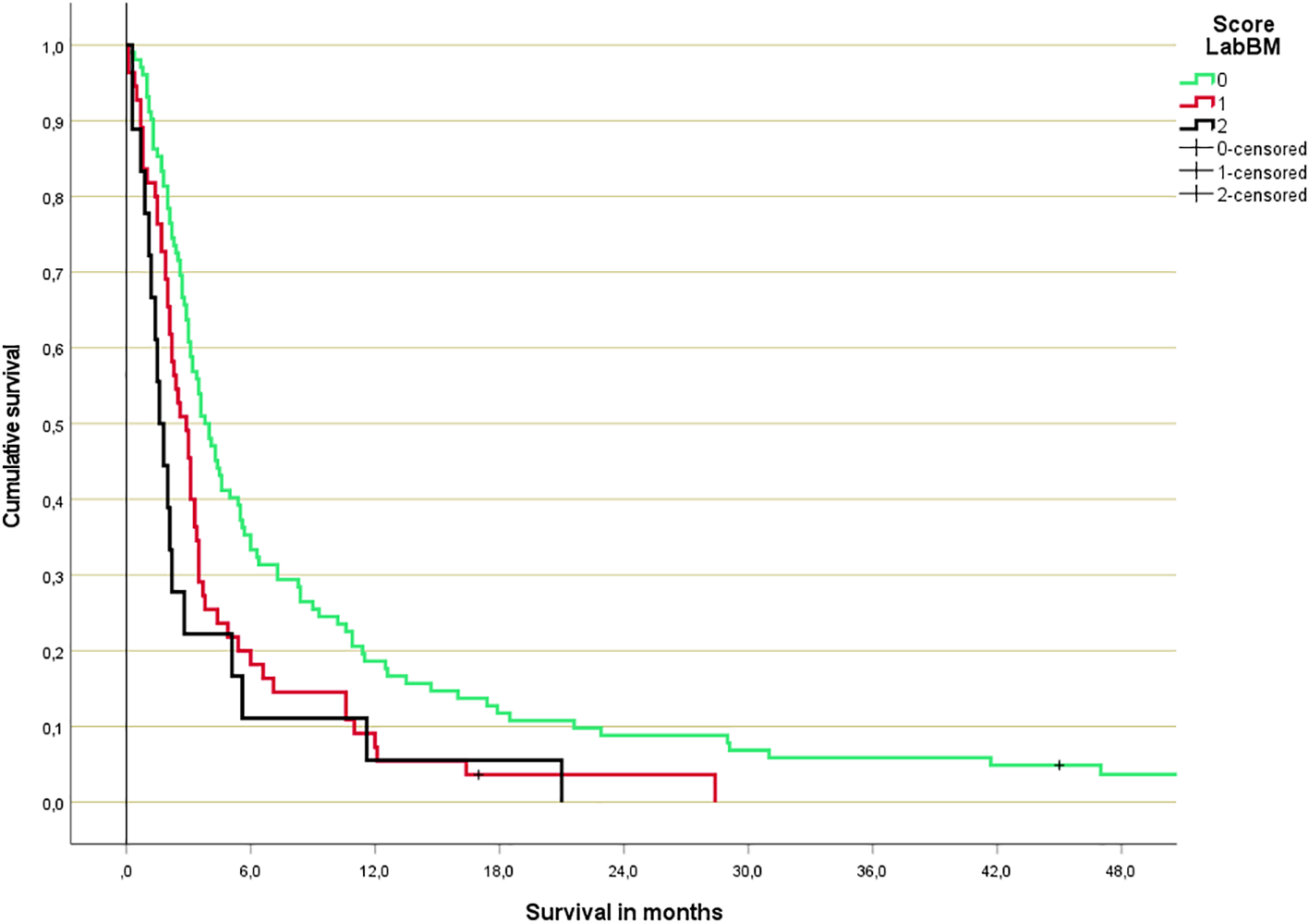
Original LabBM score 3-tiered survival prediction (Kaplan-Meier analysis; p<0.001 over all strata; median in months: 3.8 (0-1 points), 2.8 (1.5-2 points), 1.6 (2.5-3.5 points); n=102, 55, 18).

**Figure 6:**
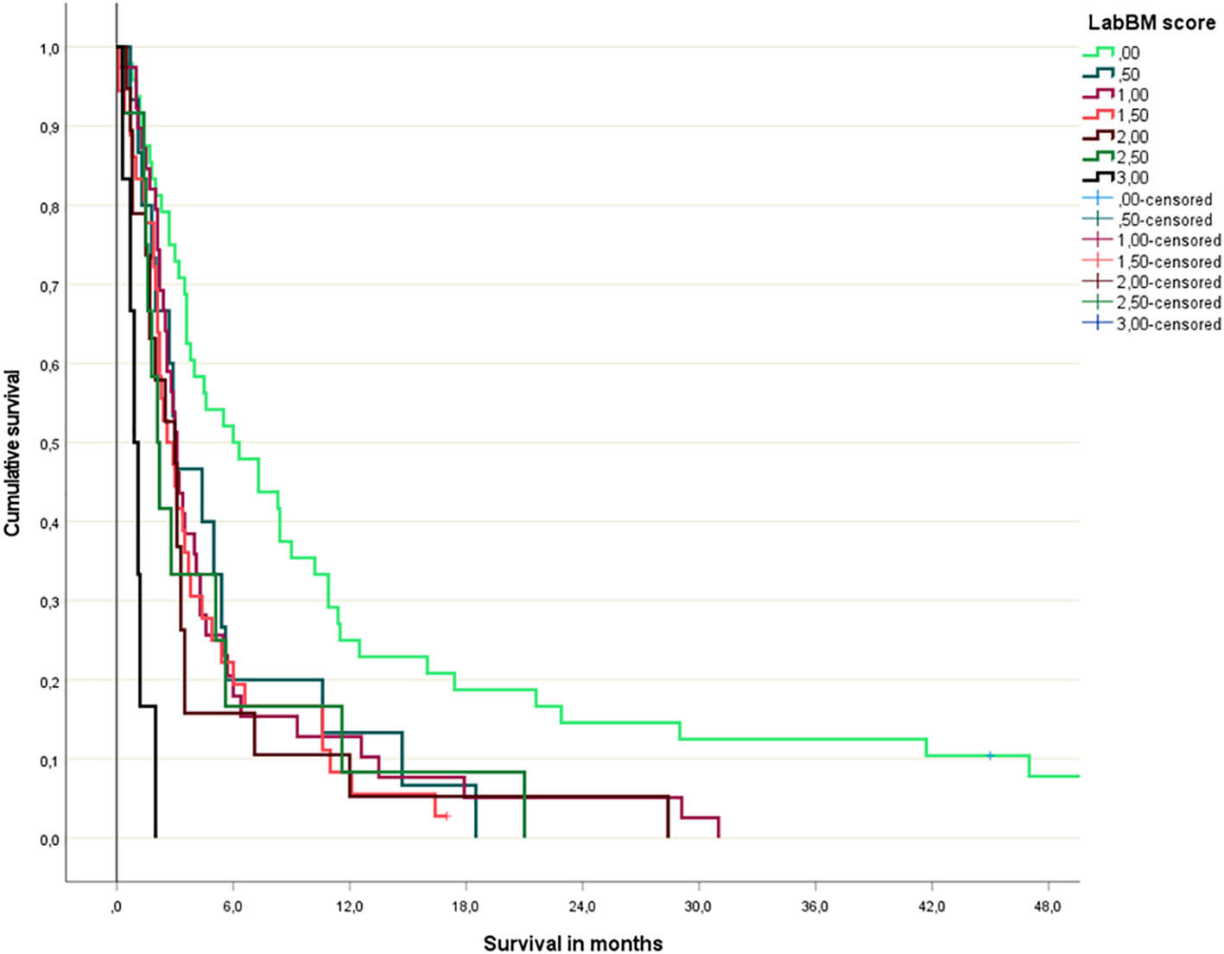
Original LabBM score (Kaplan-Meier analysis; p=0.04 or better for all comparisons involving patients with 0 points; p=0.003 or better for all comparisons involving patients with 3 points).

The multivariate Cox regression analysis with all 5 blood tests (normal/abnormal albumin, CRP, platelets; 3-tiered hemoglobin and LDH) is shown in **Table 2**. All 3 significant predictors of survival were employed to assign a modified LabBM score, which is displayed in **Table 3**. **Figure 4** shows the Kaplan-Meier survival curves (4 strata were possible) and **Figure 5** shows the original three-tiered LabBM score. The original LabBM score with 5 blood tests could not be converted into a 4-tiered score, because all intermediate survival curves were overlapping, as shown in **Figure 6**. Both versions were identical in terms of patients with 0 points (n=48, median survival 6 months) and patients with highest point sum (n=6 with 3 points (original), n=6 with 2 points (modified), median survival 0.9 months).

**Table 2:** Multivariate forward stepwise Cox regression analysis.

Blood test	B	SE	Wald	Exp(B)	p-value
Lactate dehydrogenase (3-tiered)	-.247	.07	12.1	.78	<0.001
Hemoglobin (3-tiered)	-.246	.08	9.2	.78	0.002
C-reactive protein (dichotomized)	-.386	.16	5.7	.68	0.017
Albumin (dichotomized)					0.296
Platelets (dichotomized)					0.530

**Table 3:** Modified LabBM score (point sum 0: 3 normal blood tests).

Blood test	Abnormal	Highly abnormal	
Lactate dehydrogenase (3-tiered)	0.5 points	1 point	
Hemoglobin (3-tiered)	0.5 points	1 point	
C-reactive protein (dichotomized)	0.5 points	not applicable	
Point sum	Number	% 6-mo survival	Median survival, mo
0	48	50	6.0
0.5-1	107	23	3.1
1.5	19	5	2.0
2	6	0	0.9

## 4 Discussion

The purpose of the present proof-of-principle study was to test the role of non-dichotomized blood test results in comparison to the original version of the LabBM score with dichotomized blood tests. A homogeneously treated patient population with few censored events was selected, which was characterized by poor prognostic features such as multiple brain metastases and typically also extracranial metastases (85% of patients). Due to these features, WBRT was selected as primary modality, while our institution preferred radiosurgery for patients with better prognosis.

We showed that analyses of abnormal blood tests as continuous variables, i.e. the truly observed distribution, are feasible, but not uniformly associated with a gain in prognostic information. For albumin and CRP, dichotomized values continued to represent the preferred strategy. For LDH and hemoglobin, no more than 3 strata were needed to separate the cohort in the best possible fashion. Unfortunately, detailed analyses of low platelet counts were not possible. The multivariate analysis demonstrated that changes in variable stratification, i.e. 3 strata rather than 2, led to changes of the resulting score components, e.g., reduction to 3 significant predictors of survival (LDH, hemoglobin, CRP). The modified LabBM score was both, easier to assign (albumin and platelets were no longer needed) and better able to discriminate or subclassify intermediate class patients, i.e. those not having the minimum or maximum point sum.

It is not justified to claim that the modified LabBM score represents the best possible score that can be developed with the 5 underlying blood tests, because of several limitations of this preliminary study. Small subgroups, unknown test results in some patients, and the risk of overfitting statistical models in the absence of validation strategies have to be mentioned here. However, we felt that a moderately sized study might represent a useful first step before one allocates lots of resources to a large analysis, without knowing that positive signals support this avenue of research. Large-scale analyses are also necessary to study the potential for nomogram development, based on very granular blood test differences, which eventually might provide more information than the present categories.

The study confirmed the previously reported limited survival after primary WBRT, which has resulted in increasing utilization of radiosurgery also in patients with multiple brain metastases (16–18). Due to its relatively low biologically effective radiation dose, WBRT is not expected to provide long-lasting local control of visible brain metastases larger than few millimeters. However, long-lasting control becomes increasingly important if systemic therapy improves and, thus, provides durable extracranial disease stabilization. Utilization of the LabBM score, or blood biomarkers in a broader sense, renders comprehensive radiological assessment of primary tumor control and extracranial metastases unnecessary and is a low-cost intervention. Implementation of the LabBM score in clinical routine is feasible and might improve decision making and prediction of short survival. Possible modifications of the score beyond the current strategy have already been proposed and include assessment of tumor markers such as carcinoembryonic antigen (19).

## Data availability statement

The raw data supporting the conclusions of this article will be made available by the authors, without undue reservation.

## Ethics statement

Ethical review and approval was not required for the study on human participants in accordance with the local legislation and institutional requirements. The patients/participants provided their written informed consent to participate in this study.

## Author contributions

CN: collected the related data, contributed to analysis of the data, investigated the study results, and wrote the manuscript. NA: interpreted data, and reviewed the manuscript. AG: interpreted data and reviewed the manuscript. All authors contributed to the article and approved the submitted version.
